# Phenotypic and fine genetic characterization of the *D *locus controlling fruit acidity in peach

**DOI:** 10.1186/1471-2229-9-59

**Published:** 2009-05-15

**Authors:** Karima Boudehri, Abdelhafid Bendahmane, Gaëlle Cardinet, Christelle Troadec, Annick Moing, Elisabeth Dirlewanger

**Affiliations:** 1 INRA, UR0419, Unité de Recherches sur les Espèces Fruitières, Centre de Bordeaux, BP 81, F-33140 Villenave d'Ornon, France; 2 INRA-CNRS, UMR1165 Unité de Recherche en Génomique Végétale (URGV), 2 rue Gaston Crémieux, F-91057 Evry, France; 3 INRA – UMR619 Fruit Biology, INRA, Université de Bordeaux 1, Université de Bordeaux 2, BP 81, F-33140 Villenave d'Ornon, France; 4 Metabolome-Fluxome Pole, IFR103 BVI, BP 81, F-33140 Villenave d'Ornon, France

## Abstract

**Background:**

Acidity is an essential component of the organoleptic quality of fleshy fruits. However, in these fruits, the physiological and molecular mechanisms that control fruit acidity remain unclear. In peach the *D *locus controls fruit acidity; low-acidity is determined by the dominant allele. Using a peach progeny of 208 F_2 _trees, the *D *locus was mapped to the proximal end of linkage group 5 and co-localized with major QTLs involved in the control of fruit pH, titratable acidity and organic acid concentration and small QTLs for sugar concentration. To investigate the molecular basis of fruit acidity in peach we initiated the map-based cloning of the *D *locus.

**Results:**

In order to generate a high-resolution linkage map in the vicinity of the *D *locus, 1,024 AFLP primer combinations were screened using DNA of bulked acid and low-acid segregants. We also screened a segregating population of 1,718 individuals for chromosomal recombination events linked to the *D *locus and identified 308 individuals with recombination events close to *D*. Using these recombinant individuals we delimited the *D *locus to a genetic interval of 0.4 cM. We also constructed a peach BAC library of 52,000 clones with a mean insert size of 90 kb. The screening of the BAC library with markers tightly linked to *D *locus indicated that 1 cM corresponds to 250 kb at the vicinity of the *D *locus.

**Conclusion:**

In the present work we presented the first high-resolution genetic map of *D *locus in peach. We also constructed a peach BAC library of approximately 15× genome equivalent. This fine genetic and physical characterization of the *D *locus is the first step towards the isolation of the gene(s) underlying fruit acidity in peach.

## Background

Peach [*Prunus persica *(L.) Batsch] belongs to the *Spiraeoideae *subfamily of the *Rosaceae *[1]. The *Prunus *genus is characterized by species producing drupes as fruit, and contains a significant number of economically important fruit tree species such as almond (*Prunus dulcis *(Mill.)), apricot (*Prunus armeniaca *L.), sweet cherry (*Prunus avium *L.), sour cherry (*Prunus cerasus *L.) and plum (*Prunus domestica *L.).

Compared to other tree species, peach has a relative small diploid genome (290 Mb) [2], and a short juvenile phase (two to three years). Therefore, peach is considered as a model species for *Rosaceae *family and a physical map of its genome has been initiated [3].

Among fruit producing rosaceous crops, peach is the second most important fruit crop in Europe after apple and the third worldwide (FAOSTAT: http://faostat.fao.org/). However, the consumption of peaches and nectarines is stagnant due to the low quality of fruits that are harvested at an immature stage for storage and shipment reasons [4]. One of the major objectives for peach breeders is to find the right compromise between quality and immaturity at harvest [5]. The variation in fruit quality at harvest involves a large number of interrelated factors [6] among which organic acid and soluble sugar contents and composition are major determinants [7]. In ripe peach fruit, malic and citric acids are the predominant organic acids, while quinic acid accumulates in lower amounts [8,9]. Moreover, the major soluble sugars are sucrose, fructose, glucose and sorbitol [9,10]. Sucrose is the predominant soluble sugar at maturity while sorbitol accumulates at very low levels.

In peach, the *D *locus (*D *is for 'Doux' meaning 'sweet' in French) was described as dominant and controlling the 'low-acid' character of fruit [11,12]. Based on previous segregation analyses of an F_2 _population (JxF) obtained from a cross between 'Ferjalou Jalousia^®^' low-acid variety and 'Fantasia' normally-acid variety, the *D *locus was mapped on linkage group 5 [13]. It is co-localized with major QTLs for pH, titratable acidity (TA), organic acids concentration and with small QTLs for sugars concentration [14]. Low-acid peach fruit is characterized by reduced contents of malic and citric acids [9], which, however, cannot be explained just by the reduced expression or activity of phospho*enol*pyruvate carboxylase (PEPC) [15], a key enzyme involved in malate synthesis. 'Ferjalou Jalousia^®^' fruit has half the concentration of malic acid and one-fifth that of citric acid of 'Fantasia' variety [9]. Using the candidate gene approach, 18 genes involved in organic acid synthesis, degradation or vacuolar storage were studied [16,17]. Expression analyses in fruit of six selected candidate genes did not show a clear difference between the normally-acid and low-acid varieties [17]. The genes showing a modification of their expression in the low-acid fruit compared to the normally-acid fruit were the tonoplastic proton pumps *PRUpe;AtpvA1*, *PRUpe;Vp2*, and to a lesser extent *PRUpe;Vp1*. *PRUpe*;*Vp1 *and *PRUpe*;*Vp2 *at citric acid peak and maturity, and *PRUpe;AtpvA1 *during cell division showed higher expression in the fruit of the low-acid variety ('Ferjalou Jalousia^®^'). However, none of these candidate genes were located on linkage group 5, excluding their direct role in the control of acid content by the *D *locus [17]. More recently, in the European ISAFRUIT Integrated Project http://www.isafruit.org/Portal/index.php, several candidate genes involved in fruit quality were selected and tested on the JxF F_2 _mapping population. However, none of them was located in the region of the *D *locus (Dirlewanger E., manuscript in preparation)

Low-acid varieties have already been described in apple [18], tomato [19], grape [20] and several *Citrus *species [21]. In apple, a non-acid mutant from the 'Usterapfel' variety showed a content in malic acid ten times less than the normally-acid one [22].

The high level of malic acid was reported to be controlled by the dominant *Ma *allele [23] suggesting that *Ma *and *D *act at different physiological control points. A cDNA-AFLP analysis, coupled with a bulk segregant analysis (BSA) was recently used to screen genes differently expressed between low- and high-acid apple fruits [24]. The authors reported the isolation of a cDNA whose expression could only be detected in low-acid fruit at an early stage of fruit development. Nevertheless, this cDNA showed no homology with any sequences in public databases. Moreover, the *Ma *and *D *loci are not located on homologous chromosomes: *Ma *is located on linkage group 16 in *Malus *[25], homologous to linkage group 1 in *Prunus *[26] and *D *is located on linkage group 5 in *Prunus *which is homologous to linkage groups 6 and 14 in *Malus *[27]. For *Citrus*, the low level of citric acid is controlled by a recessive gene named *acitric *[28]. Fruit acidity in *Citrus *seems to be linked to the capacity to accumulate citric acid into the vacuole. Low-acid varieties accumulate low amount of citric acid probably because it is exported out from the vacuole [29,30]. Two candidate genes such as acid invertase and cytoplasmic isocitrate dehydrogenase were identified to be differentially expressed between acid and low-acid *Citrus *[30]. Fruit acidity can also be controlled by several chromosome regions as in tomato where several QTLs for titratable acidity and pH were identified [31,32] and several candidate genes were proposed [33]. However, to date the mechanism(s) of the genetic control of fruit acidity remains to be elucidated.

In order to identify genes of interest, candidate gene approach can be used when assumptions can be made regarding the biological function of the gene [34]. This approach was successfully undertaken for several fruit traits including anthocyanin content for which the biosynthesis pathway and regulating genes were well known [35] and cell wall degradation where implicated genes were identified in other species [36]. However, to isolate agronomically important and botanically relevant genes with unknown function and where no clear hypothesis can be made, chromosome landing seems the main strategy by which map-based or positional cloning could be applied [37]. The complexity of organic acids metabolic pathways as well as the difficult understanding of the regulation of their transporters and channels and related proton pumps [38,39] has hampered, so far, the identification of the gene(s) associated to the *D *locus using a candidate gene approach. Thus, in order to understand the molecular and physiological bases of this trait, a positional cloning strategy was initiated and a fine map of the *D *locus has been constructed. To identify the gene(s) underlying acidity control at the *D *locus, the first step was to construct a fine map of the *D *locus. The aims of the present work were: (1) the characterization of the fruit acidity trait, (2) the increase of the number of markers tightly linked to the *D *locus, (3) the conversion of the nearest markers into Sequence Characterized Amplified Region (SCAR) and Cleaved Amplified Polymorphic Sequence (CAPS) markers, (4) the construction of a high-resolution genetic map of this locus and definition of the position of the *D *locus with new recombinant individuals phenotyped, and (5) the evaluation of the genetic distance/physical distance ratio around the *D *locus using a BAC library.

## Results

### Fruit acidity characterization

**Figure 1:**
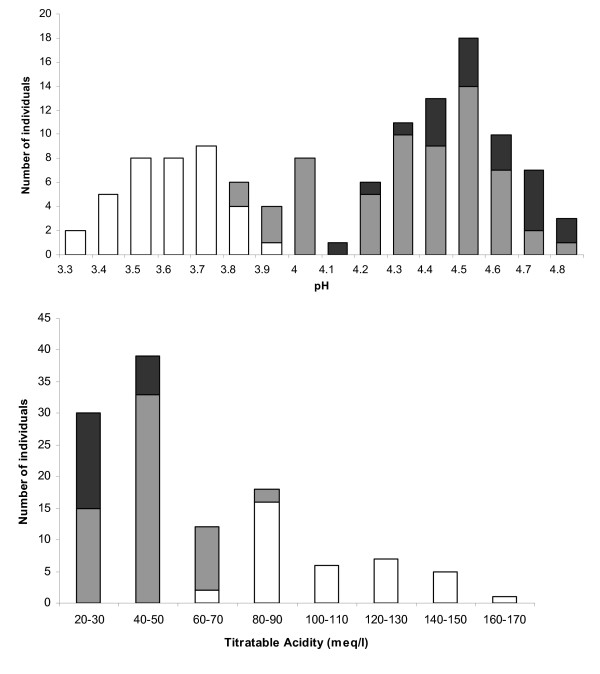
**Frequency distribution of pH and titratable acidity of fruit juice**. Mean values observed in 2007 are represented. Distribution of F_2 _individuals, having sfruit at maturity, used for the genetic linkage map. White, black and grey bars indicate homozygous genotypes for 'Fantasia' allele, 'Ferjalou Jalousia^®^' allele and heterozygous, respectively.

Among the 208 individuals used for the genetic linkage map, only 151 trees producing fruit were phenotyped for pH and titratable acidity and were classified into three subgroups corresponding to the three genotypes: homozygous for 'Ferjalou Jalousia^®^' allele (JJ) and for 'Fantasia' allele (FF), and heterozygous (JF) at the targeted locus (Fig. 1). A significant difference (Student's t-test, *P *< 0.01) was observed for pH and TA for the comparisons of JJ and JF genotypes, FF and JF genotypes, and JJ and FF genotypes (pH mean values for JJ = 4.57, JF = 4.36, FF = 3.63; TA mean values JJ = 36.5, JF = 48.2, FF = 109.7 meq/l) suggesting that the *D *allele is partially dominant. Homozygous JJ genotypes showed values higher than 4.12 for pH and lower than 51.9 meq/l for TA. On the opposite, pH and TA values for homozygous FF genotypes were respectively lower than 3.93 and higher than 65.6 meq/l. The pH of heterozygous JF genotypes ranged from 3.80 to 4.87 and TA ranged from 28.1 to 90.0 meq/l. Thus, normally-acid phenotypes that correspond exclusively to genotypes FF showed pH value lower than 3.8 and TA value higher than 100 meq/l while low-acid phenotypes corresponding to genotypes JJ or JF showed pH values higher than 4.0 and TA value lower than 60 meq/l. These results indicate that individuals with intermediate acidity (pH values between 3.8 and 4.0 and TA values between 60 and 100 meq/l) can be either homozygous dd or heterozygous Dd at the *D *locus and therefore, they cannot be reliably classified into normally-acid or low-acid phenotype.

### Identification and mapping of AFLP markers linked to the *D *locus

**Figure 2:**
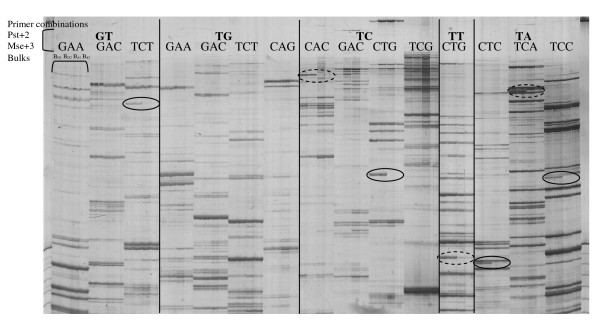
**AFLP markers detecting polymorphisms between low-acid bulks and normally-acid bulks revealed on polyacrylamide gel**. AFLP markers showing a band in low-acid bulks (B_D1 _and B_D2_) but not in normally-acid (B_d1 _and B_d2_) bulks are surrounded with a continuous line. AFLP markers showing a faint band in B_d1 _and B_d2 _bulks, not selected for genetic mapping, are surrounded with a dotted line.

Among the 1,024 primer combinations tested, 960 provided readable amplification products. Thirty to 90 bands were observed on AFLP gels per primer combination with a size range from 60 to 1,000 bp, but only 6.5% of the bands were polymorphic between the 'Ferjalou Jalousia^®^' and 'Fantasia' parents. Markers whose bands were present in B_D1 _and B_D2 _bulks and absent from B_d1 _and B_d2 _bulks were potentially linked to the *D *locus (Fig. 2). A total of 34 markers were identified as putatively linked to the *D *locus (Table 1). Nineteen primer combinations each revealed only one *D*-linked marker, six primer combinations produced two *D*-linked markers (pGC-AGG, pCA-GCG, pTC-CAC, pCA-ACC, pCA-TCC and pAA-ACA) and one primer combination revealed three *D*-linked markers (pGC-TCT). As expected, all 34 AFLP markers were mapped on linkage group 5. AFLP markers close to the *D *locus (within 22 cM) were clearly polymorphic between bulks. AFLP markers mapped further away (beyond 27 cM) were polymorphic markers between bulks but with a very faint band for "d" bulks. Fourteen AFLP markers were located within the first 10 cM containing the *D *locus.

**Table 1:** AFLP markers mapped on linkage group 5 (LG5) based on 208 JxF F_2 _individuals

AFLP marker_size in bp*_	Position on LG5 (cM from the top)	Selection for conversion into SCAR	SCAR marker
pAC-AAC_402J-412F_	0	Yes	D-Scar1
pGG-TAC_215J-221F_	0	Yes	D-Scar2
pGC-AGG_430J-450F_	0.7	Yes	D-Scar0
pTC-CTG_470J_	0.7	Yes	D-Scar7
pGT-TTG_188J_	0.7	Yes	Monomorphic
pCA-GCG_149F_	0.9	Yes	Monomorphic
pTC-GTA_218F-219J_	1.8	Yes	D-Scar3
pCA-GCG_132J_	3.3	Yes	Monomorphic
pTC-CAC_206J_	4.1	No	
pTG-TGG_470J_	4.9	Yes	D-Scar6
pCA-GTA_390J_	5.6	No	
pCA-ACC_168J_	9.1	No	
pGC-TCT_232F_	9.2	No	
pGG-TGA_380J_	10	No	
pCC-AGT_223J_	11.7	No	
pTA-TCC_470J-475F_	11.7	No	
pCC-GAA_202J_	12	No	
pCT-CAT_203J_	12.2	No	
pAG-GTA_202J_	12.4	No	
pTA-GTG_600J_	15.2	No	
pGC-TCT_380J_	16.2	No	
pGC-TCT_370F_	17.4	No	
pGT-TCT_550J_	21.8	No	
pAT-TTC_360J_	22	No	
pTA-CTC_317J_	22.2	No	
pCA-TCC_400J_	27	No	
pTC-CAC_350F_	30.2	No	
pAA-ACA_253F-255J_	39.8	No	
pCA-GAC_158J_	40.7	No	
pCA-ACC_254J_	46.6	No	
pGC-AGG_500J_	47.8	No	
pCT-ATC_220J_	63.6	No	
pAA-ACA_400J_	68	No	
pCA-TCC_370J_	78.1	No	

* Allele sizes for both parents are indicated for codominant markers, while only allele size for one parent is indicated for dominant markers. ^J ^'Ferjalou Jalousia^®^', ^F ^'Fantasia'.

### Conversion of AFLP markers into SCAR and CAPS markers

**Figure 3:**
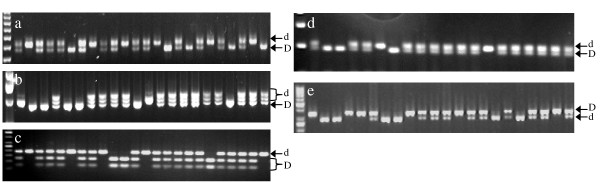
**F_2 _individuals screened with SCAR markers on agarose gel**. (a) D-Scar0 on 3% agarose gel (b) D-Scar1 on 2% agarose (c) D-Scar3 digested with *Acc*I, on 2% agarose gel (d) D-Scar6 on 3% agarose gel (e) D-Scar7 digested with *Mse*I, on 3% agarose gel. Specific band for each allele is indicated (d for normally-acid and D for low-acid).

Nine AFLP markers linked to the *D *locus were converted into simple codominant PCR-based markers. Four of them were codominant markers and five were dominant markers (Table 1). The codominant AFLP markers (pGC-AGG _430J-450F_, pAC-AAC_402J-412F _and pGG-TAC_215J-221F_) were successfully converted into SCAR markers (D-Scar0, D-Scar1 and D-Scar2) and were confirmed as codominant markers (Table 2, Fig. 3). The codominant AFLP markers pTC-GTA_218F-219J _revealed a deletion of one nucleotide in 'Fantasia' compared to 'Ferjalou Jalousia^®^'. After sequencing the two alleles, three single nucleotide polymorphisms (SNPs) were detected; one of them was revealed after digestion with the restriction enzyme *Acc*I and directly observed on agarose gel. This codominant Cleaved Amplified Polymorphism Sequence (CAPS) marker was named D-Scar3 (Fig. 3).

For the dominant AFLP markers, primers were designed from the sequences of individuals carrying the *D *allele. They were then tested on 'Ferjalou Jalousia^®^', 'Fantasia' and on the F_1_-JF:21 hybrid used to construct the F_2 _mapping progeny. The comparison of the sequences obtained for the pTG-TGG_470J _marker revealed a deletion of six nucleotides in 'Fantasia' as compared to 'Ferjalou Jalousia^®^' sequence. This AFLP marker was then transformed into a codominant SCAR marker (D-Scar6) (Fig. 3). For pTC-CTG_470J_, the sequencing of the two alleles revealed one SNP. The alleles can be discriminated by digesting the PCR products with *Mse*I. This CAPS marker was called D-Scar7 (Fig. 3). For the three dominant AFLP markers pGT-TTG_188J_, pCA-GCG_149F _and pCA-GCG_132J_, no polymorphism was detected.

**Table 2:** SCAR and CAPS markers developed from AFLP fragments linked to the D locus

AFLP marker_size in bp_^1^	SCAR marker	Primer sequence (5'-3')	Size^1 ^(bp)	P^2^	Annealing temp. (°C)	Enzyme
pGC-AGG_430J-450F_	D-Scar0	*F *GTGCACAGCTATCTCCTTTC	160 (J)	SSR	52	no
		*R *CTCATGGCAACAACATATTC	175 (F)			
pAC-AAC_402J-412F_	D-Scar1	*F *GGGATGTGGGTATGTCGTA	345 (J)	SSR	55	no
		*R *ACAAGGAGGAAGCTCTGG	364 (F)			
pGG-TAC_215J-221F_	D-Scar2	*F *CCTTACGTCTACGACGACAAC	142 (J)	InDel	54	no
		*R *TGAGTCCTGAGTAATACTGTTCATGTG	148 (F)			
pTC-GTA_218F-219J_	D-Scar3	*F *GTTGACATGAAACAAATGACATTG	180 (J, F)	SNP	52	*Acc*I
		*R *CAGTCGTTCTTGTAGTTCACATGC				
pTG-TGG_470J_	D-Scar6	*F *CATGGCCCCATCTTTTCAC	92 (J)	InDel	55	no
		*R *GACCAGTTGCATCTCATTCATATTGG	98 (F)			
pTC-CTG_470J_	D-Scar7	*F *CTGGTCATCTACCGTCTC	334 (J, F)	SNP	55	*Mse*I
		*R *TCCAACTCCAAGGCTTGC				

^1 ^(J) 'Ferjalou Jalousia^®'^, (F) 'Fantasia', ^2 ^Polymorphism observed.

The six polymorphic SCAR markers were subsequently used to genotype the 208 individuals of the genetic linkage map; it confirmed that their localization was the same as AFLP markers (data not shown).

Furthermore, considering the total size of the obtained sequences (2,711 bp), this analysis revealed seven SNPs. Based on these results the frequency of the SNPs at the vicinity of the *D *locus was estimated to 2.6 SNPs per kb.

### High resolution mapping of the *D *locus

**Figure 4:**
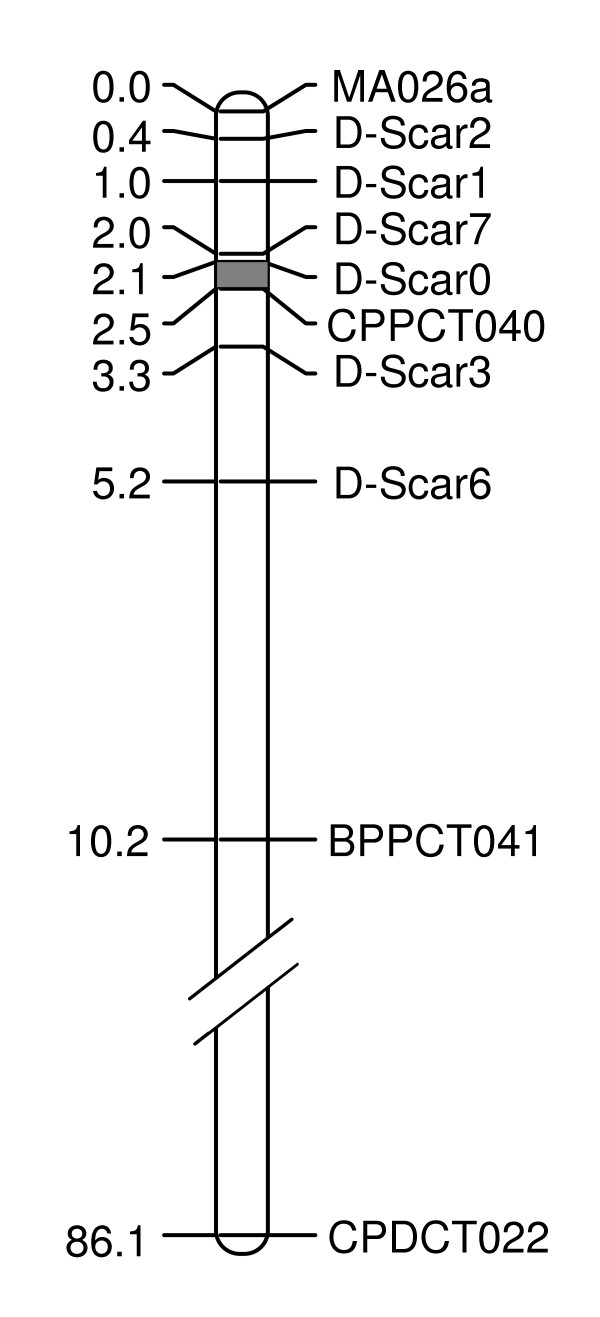
**Fine genetic map of the *D *locus on the distal end of linkage group 5**. SSR and SCAR markers are on the right and genetic distances are indicated on the left from the top (cM) and based on the analyses of 1,718 F_2 _individuals. The grey part corresponds to the position of the *D *locus.

The fine mapping of the *D *locus was performed in two steps using the six SCAR markers described in the present study and three SSR markers MA026a, BPPCT041 and CPPCT040 already mapped to the proximal end of the linkage group 5 [13,26]. The first step was to genotype the 1,718 individuals from the seven segregating populations with three SCAR markers (D-Scar0, D-Scar2 and D-Scar6) and two SSR markers (MA026a and BPPCT041) spanning a region of 10.2 cM around the *D *locus (Fig. 4). A total of 308 individuals were found to have at least one recombination event between the farthest markers, MA026a and BPPCT041. The second step was to genotype the resulting 308 recombinant individuals with the three other SCAR markers (D-Scar1, D-Scar3 and D-Scar7) and CPPCT040 which were located within the spanned region. According to the recombination events between these nine markers, it was possible to determine the precise marker order on linkage group 5 (Fig. 4).

**Figure 5:**
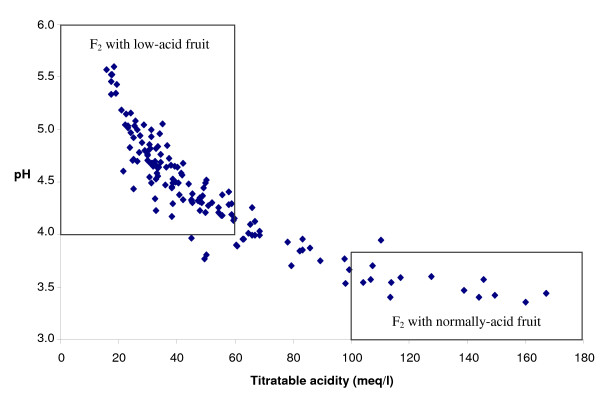
**Biplot of pH and titratable acidity of fruit juice**. Mean values observed in 2007 are represented. pH and TA biplot for F_2 _individuals recombining between MA026a and BPPCT041, phenotyped among the 1,718 F_2 _individuals. Individuals not included in squares are considered as intermediate.

For the 149 recombinant individuals which produced fruits in 2007 and 2008, TA mean values varied from 15 to 167 meq/l and pH mean values ranged from 3.36 to 5.59 (Fig. 5). Among these recombinants, 110 individuals were classified as producing low-acid fruit, 12 were identified as producing normally-acid fruit and 27 were considered as intermediate and were therefore, not classified. Among the individuals producing low-acid fruit, only 40 individuals recombining from heterozygous (Dd) to homozygous (dd) were informative. Then, only 52 recombinant individuals with extreme values for pH (from 3.36 to 3.68 for individuals with normally-acid fruit and from 4.20 to 5.55 for individuals with low-acid fruit) and TA (from 104 to 167 meq/l for individuals with normally-acid fruit and from 17 to 56 meq/l for individuals with low-acid fruit) were used to identify the position of the *D *locus. Among the 52 recombinant individuals so selected, 36 recombinant individuals between CPPCT040 and BPPCT041 indicated that the *D *locus was located upper than CPPCT040, while fourteen other individuals recombining between MA026a and D-Scar7 proved that the *D *locus was not localized between MA026a and D-Scar1 (Table 3). Two phenotyped individuals recombining between D-Scar0 and CPPCT040 reduced the interval containing the *D *locus: S5848-228 showed that the *D *locus was located upper than CPPCT040 while S6422-237 demonstrated that it was located below D-Scar0 (Table 3). Therefore, it can be concluded that the *D *locus is localized in a 0.4 cM interval between D-Scar0 and CPPCT040 (Fig. 4).

**Table 3:** Genotypes and phenotypes of F_2 _recombinant individuals for nine markers framing the *D *locus

Individual	P^1^	Genotype^2 ^(G)									No of F_2 _*
			
		MA026a	D-Scar2	D-Scar1	D-Scar7	D-Scar0	CPPCT040	D-Scar3	D-Scar6	BPPCT041	
S8220-1186	[D]	**H**	**H**	**H**	**H**	**H**	**H**	**H**	**H**	F	15
S8220-1321	[d]	F	F	F	F	F	F	F	F	**H**	3
S5848-350	[D]	**H**	**H**	**H**	**H**	**H**	**H**	**H**	F	F	11
S8220-1090	[d]	F	F	F	F	F	F	F	**H**	**H**	3
S5848-332	[D]	**H**	**H**	**H**	**H**	**H**	**H**	F	F	F	2
S6422-022	[d]	F	F	F	F	F	F	**H**	**H**	**H**	2
S5848-228	[D]	**H**	**H**	**H**	**H**	**H**	F	F	F	F	1
S6422-237	[D]	F	F	F	F	F	**H**	**H**	**H**	**H**	1
S8220-2037	[d]	**H**	**H**	**H**	F	F	F	F	F	F	1
S8220-1188	[D]	F	F	F	**H**	**H**	**H**	**H**	**H**	**H**	7
S6361-020	[d]	**H**	**H**	F	F	F	F	F	F	F	2
S5848-147	[D]	F	F	**H**	**H**	**H**	**H**	**H**	**H**	**H**	1
S6422-452	[d]	**H**	F	F	F	F	F	F	F	F	1
S8220-1045	[D]	F	**H**	**H**	**H**	**H**	**H**	**H**	**H**	**H**	2

^1 ^P: Phenotype, [d]: normally acid fruit, [D]: low acid fruit, ^2 ^F: homozygous for the 'Fantasia' allele (dd), H: heterozygous highlighted in bold (Dd), * Number of F_2 _individuals having the same phenotype and genotype

### Evaluation of the physical/genetic distance ratio around the *D *locus using a new BAC library

The peach BAC library produced from F_1_-JF:21 DNA contained about 52,000 clones. Based on the analyses of a subset of clones, the average insert size was estimated at 90 kb, ranging from 50 to 130 kb. According to these preliminary results the covering of this BAC library was estimated at 15–16 × the peach haploid genome. D-Scar0 and D-Scar7 were used to screen the BAC library and four positive clones were found with D-Scar0 and 12 with D-Scar7. Three of the four positive clones with D-Scar0 were found to be also positive when screened with D-Scar7. The ratio between the genetic and physical distances was estimated using markers defined from BACend sequences of a positive clone common to both D-Scar0 and D-Scar7 (Table 4). A distance of 0.6 cM was estimated between the two CAPS markers F109-15-06 and R109-15-06 (Table 4) derived from the BACend sequences of one BAC containing an insert of 150 kb. This results in 1 cM corresponding to 250 kb in the region of the *D *locus.

**Table 4:** Markers developed from BACend sequences of a positive BAC clone with both D-Scar0 and D-Scar7

BACend marker	Primer sequence (5'-3')	Size* (bp)	Polymorphism	Annealing temp. (°C)	Enzyme
F109-15-06	*F *GTAGGATGAACTCAAAGGTG	570 (J, F)	SNP	52	*Tsp509*I
	*R *GTTGGTAATGACACTGGCTA				
R109-15-06	*F *GTGGACTTCATCCCATCTAC	540 (J, F)	SNP	54	*Hinc*II
	*R *GGTCCAGAAGATGATGCAC				

* (J) 'Ferjalou Jalousia^®^', (F) 'Fantasia'

## Discussion

We describe in this paper major steps towards the cloning of the gene(s) controlling fruit acidity in peach, by phenotypic, genetic and physical characterization of the *D *locus.

The low polymorphism observed between 'Ferjalou Jalousia^®^' and 'Fantasia' using AFLP markers was previously reported using RFLP and SSR markers [13] and was likely the consequence of the very low genetic distance between these two parental varieties [13]. Polymorphic markers would have been considerably increased by deep sequencing methods of the AFLPs [40]. Using the classical AFLP method in combination with bulk segregant analysis, 14 AFLP markers located within the 10 cM region harbouring the *D *locus were identified and no marker was mapped to another linkage group. These results confirmed that acidity trait in peach is not complex and should be controlled by a major gene.

Sequence analysis of the AFLP markers selected for conversion into SCAR revealed, at the vicinity of the *D *locus, an SNPs frequency 3.6 fold lower than the one reported in the 'Texas' × 'Earlygold' (TxE) reference *Prunus *map derived from an almond × peach interspecific cross (Illa E., personal communication).

To more accurately position the *D *locus, it was necessary to identify individuals of the extended population that had recombination events tightly linked to the *D *locus. The strategy followed in the present work involving two successive steps (firstly the genotyping of 1,718 individuals with the *D *locus-flanking markers and secondly the analysis of the recombinant individuals with additional tightly linked markers) reduced considerably the number of individuals that needed to be genotyped and phenotypically characterized. Fruit acidity is usually evaluated by pH or TA measurements. In this work, the use of both pH and TA was essential for the characterization of fruit acidity. In addition, the definition of thresholds based on the analyses of individuals without recombination event in the MA026a-BPPCT041 interval allowed a precise characterization of the phenotype. Thus, the phenotyping of the recombinant individuals with the pH and TA threshold strategy prevented misclassification of intermediate individuals that can be either homozygous (dd) or heterozygous (Dd) for the *D *locus.

The development of tightly linked markers and the phenotyping of recombinant individuals allowed the precise localization of the *D *locus. As fruit acidity is a major selection criterion, the *D*-linked markers could be used for marker assisted selection which would allow early selection of trees with the desirable character.

To estimate the relationship between the genetic and the physical distance at the vicinity of the *D *locus we anchored a BAC clone to the genetic map. Based on this analysis the ratio was estimated to 250 kb/cM. At the peach *evergrowing *(*evg*) locus the ratio was estimated to 10 to 35 kb/cM [41]. These ratios are smaller than the estimated average ratio on the TxE *Prunus *reference map [42] which is 553 kb/cM according to the genome size [2]. This is not surprising, as the physical/genetic distance ratio is known to vary along chromosomes [43-45]. The identification of the physical/genetic distance ratio in the vicinity of the *D *locus was important for estimating the number of walks needed for cloning the *D *locus. The *D *locus was localized in a 0.4 cM interval corresponding to a physical distance of 100 kb. Thus, one or two walks with BAC clones with an insert size of 90 kb should be sufficient to identify a BAC clone harbouring the *D *locus.

Sequenced BAC clones in peach [41,46], plum, apricot [46] and pear [47] revealed a gene density of 14 to 36 genes per 100 kb genomic sequence. Thus, in the 100 kb *D *locus region 14 to 36 candidate genes are expected. To identify the *D *gene(s) among these candidate genes, the aim will be to map accurately the recombination events relative to the predicted genes. To facilitate this analysis, the PPJFH BAC library was constructed from the F_1_-JF:21 hybrid between 'Ferjalou Jalousia^®^' and 'Fantasia' to identify one BAC clone for each allele. The two orthologous BAC clones will be sequenced and annotated and genetically dissected. In further analyses, the natural variability of the candidate genes will be explored within a peach germplasm collection to associate the haplotype to the phenotypic variation. Functional studies such as reverse genetics experiments should then provide further evidence for or against their involvement in fruit acidity.

The complete sequencing of the BAC clones will provide candidate genes for the *D *locus. These candidate genes may be structural genes implicated in metabolism or transport in agreement with our existing knowledge of fruit physiology or genes with novel structural or regulatory functions. Sequencing data will also provide information about *Prunus *genome organization in this particular region, which may be compared to homologous region in other Rosaceae species and even other fruit species. Microsystems analysis across Rosaceae species will provide insight into gene order, orientation and structural rearrangements of this particular region and through comparative genomics, may contribute to improve our knowledge on evolutionary and diversification processes among this family as demonstrated for *Oryza *[48].

## Conclusion

In conclusion, the present work describes, for the first time, the fine mapping of a locus involved in a fruit quality trait on perennial plants via the chromosome landing approach. The development of tightly linked markers and the phenotyping of recombinant individuals allowed the precise localization of the *D *locus in a 0.4 cM interval corresponding to 100 kb. Using the constructed PPJFH BAC library with a mean insert size of 90 kb, one or two walks should be sufficient to identify a BAC clone harbouring the *D *locus. To our knowledge, only few fine genetic maps were realized using a large number of trees and only for resistance genes [49,50]. One of the major limitations for this strategy is the generation of a large population requiring an extended orchard maintained over several years. Our mapping population of 2,086 plants that segregates for many agronomic traits as well as the PPJFH BAC library will permit the genetic dissection of, not only the *D *locus, but also other traits such as *Af *(aborting fruit), *S *(flat/round fruit), *G *(peach/nectarine) and *Ps *(pollen sterility). This mapping population could be also exploited in any future genome sequencing project in peach where anchoring sequences or BAC contigs to the genetic map is a crucial step.

## Methods

### Plant material

The genetic linkage map was based on the segregation analyses of a peach F_2 _progeny. This progeny includes 208 individuals obtained from the selfing of a single F_1 _hybrid (F_1_-JF:21) issued from a cross between 'Ferjalou Jalousia^®^' a low-acid fruit variety and 'Fantasia' a normally-acid fruit variety. This population segregates for six Mendelian traits (low-acid/normally-acid fruit *D*, peach/nectarine *G*, flat/round fruit *S*, clingstone/freestone *F*, pollen sterility *Ps*, aborting fruit *Af*) [13] and for several characters involved in fruit quality as soluble sugar and organic acid concentrations [17]. Among the 208 individuals, 151 produced fruit at maturity while 57 produced flat fruit that fell after two months of growth and were not used in this study (Table 5).

**Table 5:** Origin of F_2 _individuals used for map construction and fine mapping of the *D *locus

Cross	F_1 _name	F_2 _name	Number of F_2 _individuals
**F_2 _used for the construction of the genetic linkage map**			
'Ferjalou Jalousia^®^' × 'Fantasia'	F_1_-JF:21	S8220	**208**
Producing fruits			151

**F_2 _additional individuals produced for the fine mapping of the *D *locus**			
'Ferjalou Jalousia^®^' × 'Fantasia'	F_1_-JF:21	S8220	418
	F_1_-JF:28	S6184	113
	F_1_-JF:104	S7133	182
'Fantasia' × 'Ferjalou Jalousia^®^'	F_1_-FJ:47	S6422	451
	F_1_-FJ:49	S6421	106
'Fantasia' × 'Fercopale Platina^®^'	F_1_-FP:10	S5848	405
'Fercopale Platina^®^' × 'Fantasia'	F_1_-PF:77	S6361	203
Total of the additional F_2 _individuals			1,878
Additional F_2 _that will have fruits according to MAS for the *Af *gene			**1,510**
**Total of the F_2 _individuals used for fine mapping**			**1,718**

**Figure 6:**
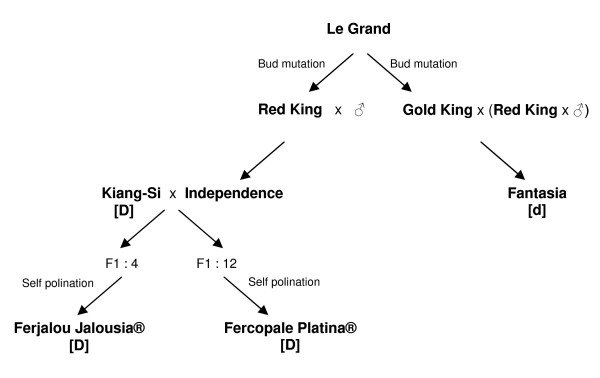
**Origin of 'Ferjalou Jalousia^®^', 'Fantasia' and 'Fercopale Platina^®^' peach varieties**. The phenotype for the *D *locus is indicated for each variety. Varieties with [D] phenotype produce low-acid fruit and varieties with [d] phenotype produce normally-acid fruit.

For the fine mapping of the *D *locus, a total of 1,878 F_2 _additional individuals were obtained from the selfing of seven different F_1 _genotypes (Table 5). Three F_1 _individuals were issued from the cross between 'Ferjalou Jalousia^®^' and 'Fantasia' (F_1_-JF:21, F_1_-JF:28, F_1_-JF:104), two from the reverse cross (F_1_-FJ:47, F_1_-FJ:49), one from a cross between 'Fantasia' and 'Fercopale Platina^®^' (F_1_-FP:10) and one from the reverse cross (F_1_-PF:77). 'Ferjalou Jalousia^®^' is homozygous for the dominant allele (DD), 'Fantasia' is homozygous for the recessive one (dd) and the seven F_1 _hybrids are heterozygous (Dd) for the *D *locus. 'Ferjalou Jalousia^®^' dominant allele is derived from 'Kiang-Si' that originated from China. 'Fercopale Platina^®^' and 'Ferjalou Jalousia^®^' shared the same common grandparents ('Kiang-Si' and 'Independence') (Fig. 6), and both had the same dominant allele for the *D *locus and produced flat peaches. According to a marker assisted selection for the *Af *gene that segregated in the 1,878 F_2 _individuals, 1,510 individuals were identified to produce fruit at maturity and were therefore genotyped. Among them, 1,084 individuals were planted in 2005 and 426 in 2006. The fine map was based on the genotyping of a total of 1,718 F_2 _individuals including the mapping population of 208 individuals and the 1,510 F_2 _additional individuals that should produce fruit at maturity (Table 5).

### Fruit acidity phenotyping

The 151 F_2 _individuals of the mapping population producing fruit and recombinant individuals among the F_2 _progenies were phenotyped in 2007 and 2008. Two harvests separated by four days were performed for each individual. For each harvest, six fruits/individual were collected at maturity stage. TA and pH analyses were measured on fruit juice by using an equal volume of juice from each fruit as described previously [14].

To avoid any misclassification of recombinant individuals, we decided to rely on the analyses of homozygous and heterozygous individuals and to define thresholds in order to distinguish individuals with low-acid fruit from those with normally-acid fruit. Individuals, without recombination event, were selected on their genotype in the MA026a-BPPCT041 interval. Student's t-test was used to compare pH and TA mean values between homozygous and heterozygous individuals.

### DNA extraction

Genomic DNA was extracted from young expanded terminal leaves. Fifteen milligrams of fresh weight were collected for each tree in 96 collection microtubes of 1.2 ml containing a tungsten carbide bead (3 mm diameter). They were ground in liquid nitrogen by using a Mixer Mill MM 300 (Retsch, Haan, Germany) for 1 min and 30 s and genomic DNA was extracted according to the method previously described [51].

### BSA-AFLP

For Amplified Fragment Length Polymorphism (AFLP) assay combined with BSA, two low-acid (*D/D *or *D/d*) DNA bulks (B_D1_, B_D2_) and two normally-acid (*d/d*) DNA bulks (B_d1_, B_d2_) were used to identify putative markers linked to the *D *locus [52]. Individuals were selected among the 208 F_2 _used for the genetic linkage map according to juice pH and TA values measured in 2002 and 2003. Equal amounts of DNA from eleven individuals from the JxF F_2 _mapping population were pooled to construct each bulk.

The AFLP technique was performed following the protocol developed by [53] with some modifications. Genomic DNA (250 ng) was digested with two restriction enzymes *Pst*I and *Mse*I in a volume of 17.5 μl. The first PCR amplification was performed with primers having no selective nucleotide and then the second PCR amplification was carried out with primers having two selective nucleotides for *Pst*I and three for *Mse*I. PCR products were mixed with an equal volume of loading buffer (95% formamide, 0.05% xylene cyanol, 0.05% bromophenol blue, 10 mM EDTA, pH 8.0). The mixture was heated for 5 min at 95°C, and then quickly cooled on ice. Each sample mixture was loaded on a 4.5% denaturing polyacrylamide gel and visualized by the silver staining system as described by [54]. Sixteen *Pst*I+2 primers and 64 *Mse*I+3 primers were tested consisting in a total of 1,024 primer combinations. Markers derived from *Pst*I+2/*Mse*I+3 primer combinations were named as pXX-YYY (X for the selective *Pst*I nucleotides and Y for the selective *Mse*I nucleotides). Subsequently, polymorphic AFLP primer combinations between bulks were used to screen the 208 F_2 _individuals of the mapping population.

### Conversion of AFLP markers into SCAR and CAPS markers

AFLP markers linked to the *D *locus were selected for conversion into PCR markers for further easy use in large-scale screening of the 1,718 individuals and BAC library. After silver staining, marker fragments of the parents and of two F_2 _individuals were picked with a tip on the dried polyacrylamide gel [55] and dissolved in 15 μl deionized water. PCR amplifications were performed using 1 μl dilution with the same conditions as the selective PCR for AFLP reaction but with primers without selective nucleotides. The products were separated on 2.0% agarose gel, purified using a MinElute^® ^PCR Purification Kit (Qiagen) and then cloned into the pGEM-T easy vector (Promega, Madison, WI, USA). Clones were sequenced by Cogenics (Meylan, France) and specific primers were designed using Primer3 software (version V.0.4.0) based on these sequences. Designed primers were then tested for PCR amplification on low-acid individuals, normally-acid individuals and also on 'Ferjalou Jalousia^®^', 'Fantasia' and the F_1 _hybrid (F_1_-JF:21). Reaction mixtures (10 μl) contained 0.2 μM of each primer, 200 μM of dNTP, 10 ng template DNA, 0.26 U of *Taq *DNA polymerase (Sigma-Aldrich), 1× PCR buffer provided with the enzyme. PCR reactions were carried out for 2 min at 94°C, followed by 38 cycles of 45 s at 94°C, 45 s at annealing temperature, 45 s at 72°C, with final elongation for 5 min at 72°C. Finally, the amplified fragments were tested for their polymorphism on a 2 to 3% agarose gel or 4.5% polyacrylamide denaturing gel.

### Segregation analysis and map construction

Each polymorphic marker was tested by a chi-square for goodness of fit to the segregation ratios 1:2:1 expected for codominant markers and 3:1 expected for dominant markers in a F_2 _population. The linkage map was constructed using the MAPMAKER/EXP V3.0 software [56]. Markers were first divided into linkage groups using a critical LOD score threshold of 5. The Kosambi function was used to convert recombination units into genetic distances.

### Fine mapping

The 1,510 additional individuals that would have fruit at maturity were used to complete the mapping population to a total of 1,718 individuals segregating for the *D *locus. These individuals were screened for five markers, two SSR markers and three new SCAR markers, spanning a large region around the *D *locus: MA026a and BPPCT041, previously mapped on JxF linkage map [13] and three AFLP markers transformed into SCAR markers. Recombinant individuals detected in this region were genotyped with three other AFLP markers transformed into SCAR markers and CPPCT040 a SSR marker mapped on the top of the linkage group 5 of 'Texas' × 'Earlygold' linkage map [26]. The phenotype of the recombinant individuals compared to the recombination point enabled the localization of the *D *locus. Among the 308 recombinant individuals detected, 149 individuals producing fruit in 2007 and 2008 were phenotyped for fruit pH and TA.

### Bacterial Artificial Chromosome (BAC) library construction

The *Prunus persica *PPJFH BAC DNA library was realized at URGV (INRA, Evry) and constructed as described previously [57]. Nuclei were isolated from 32 g of young leaves frozen in liquid nitrogen from F_1 _hybrid DNA heterozygous for all the mendelian characters segregating in the JxF cross. Restriction fragments were subjected to a double size selection in a CHEF-DRIII apparatus (Bio-Rad) by pulse field gel-electrophoresis (PFGE). The DNA from the agarose slices was electroeluted and cloned into the pIndigoBAC-5 (*Hin*d III Cloning-Ready) Vector (EPICENTRE^® ^Technologies) for ligation reactions. Competent *E. coli *DH10B cells (Invitrogen) were transformed by electroporation and transformants were selected on LB-Xgal-IPTG plates containing 12.5 μg/ml chloramphenicol. White colonies were picked using a Genetix Q-Bot and stored in 384-well microtiter plates (Genetix) at -80°C. The PPJFH BAC library was composed of 150 plates corresponding to 57,600 total BAC clones. BAC clones from each plate were mixed into pools of 384 clones (designated 'plate pools'). The BAC clones from each plate pool were resuspended into sterilized water and DNA extracted before PCR reactions. In the first step, positive plates were identified by screening the plate pools then in the second step the 16 clones of each of the 24 columns of the positive 384-well plates were pooled together and screened to identify the positive column. The third step consisted in identifying the positive BAC clone by screening the 16 clones of each positive column pool. PCR amplifications were carried out as described before for PCR experiments. Extracted DNA from BAC clones was digested with *Not*I and the digestion products were subjected to pulsed-field gel electrophoresis (PFGE) as described previously [57]: 25 μl DNA from each clone was digested by *Not*I in a 30 μl reaction mix and loaded on PFGE. Insert size was estimated using the PFGE lambda ladder (BioLabs, Frankfurt, Germany).

To associate a genetic distance to the physical distance obtained with the PFGE, primers were designed using Primer3 software (version V.0.4.0) based on two BACend sequences of a chosen positive BAC clone for two markers (D-Scar0 and D-Scar7). The primers of two markers were tested for PCR amplification on 'Ferjalou Jalousia^®^' and 'Fantasia' to identify polymorphism between the parents. Reaction mixtures and PCR conditions were done as described for SCAR markers and the amplified fragments were then sequenced. Obtained markers were used to genotype only individuals recombining between D-Scar1 and CPPCT040 framing D-Scar0 and D-Scar7.

## Authors' contributions

KB carried out the molecular genetic studies, the sequence alignment and drafted the manuscript. KB and AB conceived and designed the experiments for the BAC library. GC participated to AFLP mapping. CT carried out the BAC library pooling and extraction. KB, GC, AM and ED performed the phenotypic analysis. ED and GC participated in the genetic studies. AB and ED conceived the study and participated in its design. AB, AM and ED helped to draft the manuscript. All authors read and approved the final manuscript.

## References

[B1] Potter D Eriksson T Evans RC Oh S Smedmark JEE Morgan DR Kerr M Robertson KR Arsenault M Dickinson TA Phylogeny and classification of Rosaceae Plant Syst Evol 2007 266 1–2 5 43

[B2] Baird WV Estager AS Wells JK Estimating nuclear-DNA content in peach and related diploid species using lazer flow-cytometry and DNA hybridization J Am Soc Hortic Sci 1994 119 6 1312 1316

[B3] Zhebentyayeva TN Swire-Clark G Georgi LL Garay L Jung S Forrest S Blenda AV Blackmon B Mook J Horn R A framework physical map for peach, a model Rosaceae species Tree Genet Genomes 2008 4 4 745 756

[B4] Dirlewanger E Arús P Lörz H, Wenzel G Markers in Fruit Tree Breeding: Improvement of Peach Molecular Marker Systems in Plant Breeding and Crop Improvement 2005 55 Springer 279 302

[B5] Fideghelli C Della Strada G Grassi F Morico G The peach industry in the world: present situation and trend Acta Hort 1998 465 29 40

[B6] Genard M Bruchou C Multivariate-analysis of within-tree factors accounting for the variation of peach fruit-quality Sci Hortic 1992 52 1–2 37 51

[B7] Esti M Messia MC Sinesio F Nicotra A Conte L LaNotte E Palleschi G Quality evaluation of peaches and nectarines by electrochemical and multivariate analyses: Relationships between analytical measurements and sensory attributes Food Chem 1997 60 4 659 666

[B8] Byrne DH Nikolic AN Burns EE Variability in sugars, acids, firmness, and colour characteristics of 12 peach genotypes J Am Soc Hortic Sci 1991 116 1004 1006

[B9] Moing A Svanella L Rolin D Gaudillere M Gaudillere JP Monet R Compositional changes during the fruit development of two peach cultivars differing in juice acidity J Am Soc Hortic Sci 1998 123 5 770 775

[B10] DeJong TM Moing A Layne DR, Bassi D Carbon assimilation, partitioning and budget modeling The Peach, Botany, Production and Uses 2008 Wallingford, UK: CABI 244 263

[B11] Yoshida M Genetical studies on the fruit quality of peach varieties. I. Acidity Bull Hort Res Stn Jpn Ser A 1970 9 1 15

[B12] Monet R Transmission génétique du caractère "fruit doux" chez le pêcher. Incidence sur la sélection pour la qualité Eucarpia Fruit Section, Tree Fruit Breeding 1979; INRA, Angers, France 1979 273 276

[B13] Dirlewanger E Cosson P Boudehri K Renaud C Capdeville G Tauzin Y Laigret F Moing A Development of a second-generation genetic linkage map for peach [*Prunus persica *(L.) Batsch] and characterization of morphological traits affecting flower and fruit Tree Genet Genomes 2006 3 1 1 13

[B14] Dirlewanger E Moing A Rothan C Svanella L Pronier V Guye A Plomion C Monet R Mapping QTLs controlling fruit quality in peach (*Prunus persica *(L.) Batsch) Theor Appl Genet 1999 98 1 18 31

[B15] Moing A Rothan C Svanella L Just D Diakou P Raymond P Gaudillere JP Monet R Role of phospho*enol*pyruvate carboxylase in organic acid accumulation during peach fruit development Physiol Plant 2000 108 1 1 10

[B16] Etienne C Moing A Dirlewanger E Raymond P Monet R Rothan C Isolation and characterization of six peach cDNAs encoding key proteins in organic acid metabolism and solute accumulation: involvement in regulating peach fruit acidity Physiol Plant 2002 114 2 259 270 11903973 10.1034/j.1399-3054.2002.1140212.x

[B17] Etienne C Rothan C Moing A Plomion C Bodenes C Svanella-Dumas L Cosson P Pronier V Monet R Dirlewanger E Candidate genes and QTLs for sugar and organic acid content in peach [*Prunus persica *(L.) Batsch] Theor Appl Genet 2002 105 1 145 159 12582572 10.1007/s00122-001-0841-9

[B18] Visser T Verhaegh JJ Inheritance and selection of some fruit character of apple. 1. Inheritance of low and high acidity Euphytica 1978 27 753 760

[B19] Stevens MA Citrate and malate concentration in tomato fruits: Genetic control and maturational effects J Am Soc Hortic Sci 1972 97 655 658

[B20] Boubals D Bourzeix M Guitraud J Le Gora Chirine, variété de vigne iranienne à faible teneur en acides organiques Ann Amélior Plantes 1971 21 281 285

[B21] Cameron JW Soost RK Acidity and total soluble solids in *Citrus *hybrids and advanced crosses involving acidless orange and acidless pummelo J Am Soc Hortic Sci 1977 120 510 514

[B22] Beruter J Carbon partitioning in an apple mutant deficient in malic acid Acta Hort 1998 466 23 28

[B23] Maliepaard C Alston FH van Arkel G Brown LM Chevreau E Dunemann F Evans KM Gardiner S Guilford P van Heusden AW Aligning male and female linkage maps of apple (*Malus pumila *Mill.) using multi-allelic markers Theor Appl Genet 1998 97 1–2 60 73

[B24] Yao YX Li M Liu Z Hao YJ Zhai H A novel gene, screened by cDNA-AFLP approach, contributes to lowering the acidity of fruit in apple Plant Physiol Biochem 2007 45 2 139 145 17344054 10.1016/j.plaphy.2007.01.010

[B25] King GJ Lynn JR Dover CJ Evans KM Seymour GB Resolution of quantitative trait loci for mechanical measures accounting for genetic variation in fruit texture of apple (*Malus pumila *Mill.) Theor Appl Genet 2001 102 8 1227 1235

[B26] Dirlewanger E Graziano E Joobeur T Garriga-Caldere F Cosson P Howad W Arús P Comparative mapping and marker-assisted selection in Rosaceae fruit crops Proc Natl Acad Sci USA 2004 101 26 9891 9896 15159547 10.1073/pnas.0307937101 PMC470769

[B27] Sargent DJ Marchese A Simpson DW Howad W Fernández-Fernández F Monfort A Arús P Evans KM Tobutt KR Development of "universal" gene-specific markers from *Malus *spp. cDNA sequences, their mapping and use in synteny studies within Rosaceae Tree Genet Genomes 2009 5 133 145

[B28] Fang DQ Federici CT Roose ML Development of molecular markers linked to a gene controlling fruit acidity in *Citrus* Genome 1997 40 6 841 849 18464869 10.1139/g97-809

[B29] Albertini MV Carcouet E Pailly O Gambotti C Luro F Berti L Changes in organic acids and sugars during early stages of development of acidic and acidless *Citrus *fruit J Agric Food Chem 2006 54 21 8335 8339 17032048 10.1021/jf061648j

[B30] Albertini MV Caractérisation biochimique et moléculaire des fruits d'agrumes (*Citrus *sp.) PhD Thesis 2007 France: University of Corsica Pascal Paoli

[B31] Fulton TM Bucheli P Voirol E Lopez J Petiard V Tanksley SD Quantitative trait loci (QTL) affecting sugars, organic acids and other biochemical properties possibly contributing to flavor, identified in four advanced backcross populations of tomato Euphytica 2002 127 2 163 177

[B32] Chaib J Lecomte L Buret M Causse M Stability over genetic backgrounds, generations and years of quantitative trait locus (QTLs) for organoleptic quality in tomato Theor Appl Genet 2006 112 5 934 944 16402187 10.1007/s00122-005-0197-7

[B33] Causse M Duffe P Gomez MC Buret M Damidaux R Zamir D Gur A Chevalier C Lemaire-Chamley M Rothan C A genetic map of candidate genes and QTLs involved in tomato fruit size and composition J Exp Bot 2004 55 403 1671 1685 15258170 10.1093/jxb/erh207

[B34] Pflieger S Lefebvre V Causse M The candidate gene approach in plant genetics: a review Mol Breed 2001 7 4 275 291

[B35] Ogundiwin EA Peace CP Nicolet CM Rashbrook VK Gradziel TM Bliss FA Parfitt D Crisosto CH Leucoanthocyanidin dioxygenase gene (PpLDOX): a potential functional marker for cold storage browning in peach Tree Genet Genomes 2008 4 3 543 554

[B36] Peace CP Crisosto CH Gradziel TM Endopolygalacturonase: A candidate gene for *Freestone *and *Melting flesh *in peach Mol Breed 2005 16 1 21 31

[B37] Tanksley SD Ganal MW Martin GB Chromosome landing: a paradigm for map-based gene cloning in plants with large genomes Trends Genet 1995 11 2 63 68 7716809 10.1016/s0168-9525(00)88999-4

[B38] Kovermann P Meyer S Hortensteiner S Picco C Scholz-Starke J Ravera S Lee Y Martinoia E The Arabidopsis vacuolar malate channel is a member of the ALMT family Plant J 2007 52 6 1169 1180 18005230 10.1111/j.1365-313X.2007.03367.x

[B39] Sze H Schumacher K Muller ML Padmanaban S Taiz L A simple nomenclature for a complex proton pump: *VHA *genes encode the vacuolar H(+)-ATPase Trends Plant Sci 2002 7 4 157 161 11950611 10.1016/s1360-1385(02)02240-9

[B40] van Orsouw NJ Hogers RC Janssen A Yalcin F Snoeijers S Verstege E Schneiders H Poel H van der van Oeveren J Verstegen H Complexity reduction of polymorphic sequences (CRoPS): a novel approach for large-scale polymorphism discovery in complex genomes PLoS ONE 2007 2 11 e1172 18000544 10.1371/journal.pone.0001172 PMC2048665

[B41] Bielenberg DG Wang YE Li Z Zhebentyayeva T Fan S Reighard GL Scorza R Abbott AG Sequencing and annotation of the evergrowing locus in peach [*Prunus persica *(L.) Batsch] reveals a cluster of six MADS-box transcription factors as candidate genes for regulation of terminal bud formation Tree Genet Genomes 2008 4 3 495 507

[B42] Howad W Yamamoto T Dirlewanger E Testolin R Cosson P Cipriani G Monforte AJ Georgi L Abbott AG Arús P Mapping with a few plants: using selective mapping for microsatellite saturation of the Prunus reference map Genetics 2005 171 3 1305 1309 16118196 10.1534/genetics.105.043661 PMC1456826

[B43] Fridman E Pleban T Zamir D A recombination hotspot delimits a wild-species quantitative trait locus for tomato sugar content to 484 bp within an invertase gene Proc Natl Acad Sci USA 2000 97 9 4718 4723 10781077 10.1073/pnas.97.9.4718 PMC18299

[B44] Ballvora A Schornack S Baker BJ Ganal M Bonas U Lahaye T Chromosome landing at the tomato *Bs4 *locus Mol Genet Genomics 2001 266 4 639 645 11810236 10.1007/s004380100583

[B45] DeScenzo RA Wise RP Variation in the ratio of physical to genetic distance in intervals adjacent to the *Mla *locus on barley chromosome 1H Mol Gen Genet 1996 251 4 472 482 8709951 10.1007/BF02172376

[B46] Jung S Jiwan D Cho I Lee T Abbott A Sosinski B Main D Synteny of Prunus and other model plant species BMC Genomics 2009 10 76 19208249 10.1186/1471-2164-10-76 PMC2647949

[B47] Okada K Tonaka N Moriya Y Norioka N Sawamura Y Matsumoto T Nakanishi T Takasaki-Yasuda T Deletion of a 236 kb region around *S *_4_*-RNase *in a stylar-part mutant *S*_4_^*sm*^-haplotype of Japanese pear Plant Mol Biol 2008 66 4 389 400 18175198 10.1007/s11103-007-9277-1

[B48] Ammiraju JS Lu F Sanyal A Yu Y Song X Jiang N Pontaroli AC Rambo T Currie J Collura K Dynamic evolution of oryza genomes is revealed by comparative genomic analysis of a genus-wide vertical data set Plant Cell 2008 20 12 3191 3209 19098269 10.1105/tpc.108.063727 PMC2630430

[B49] Deng Z Huang S Ling P Yu C Tao Q Chen C Wendell MK Zhang HB Gmitter FG Jr Fine genetic mapping and BAC contig development for the citrus tristeza virus resistance gene locus in *Poncirus trifoliata *(Raf.) Mol Genet Genomics 2001 265 4 739 747 11459195 10.1007/s004380100471

[B50] Claverie M Dirlewanger E Cosson P Bosselut N Lecouls AC Voisin R Kleinhentz M Lafargue B Caboche M Chalhoub B High-resolution mapping and chromosome landing at the root-know nematode resistance locus *Ma *from Myrobalan plum using a large-insert BAC DNA library Theor Appl Genet 2004 109 6 1318 1327 15322755 10.1007/s00122-004-1749-y

[B51] Viruel MA Messeguer R Devicente MC Garciamas J Puigdomenech P Vargas F Arús P A linkage map with RFLP and isozyme markers for almond Theor Appl Genet 1995 91 6–7 964 971 10.1007/BF00223907 24169984

[B52] Michelmore RW Paran I Kesseli RV Identification of markers linked to disease-resistance genes by bulked segregant analysis: a rapid method to detect markers in specific genomic regions by using segregating populations Proc Natl Acad Sci USA 1991 88 21 9828 9832 1682921 10.1073/pnas.88.21.9828 PMC52814

[B53] Vos P Hogers R Bleeker M Reijans M Lee T van de Hornes M Frijters A Pot J Peleman J Kuiper M AFLP: a new technique for DNA fingerprinting Nucleic Acids Res 1995 23 21 4407 4414 7501463 10.1093/nar/23.21.4407 PMC307397

[B54] Bassam BJ Caetano-Anolles G Gresshoff PM Fast and sensitive silver staining of DNA in polyacrylamide gels Anal Biochem 1991 196 1 80 83 1716076 10.1016/0003-2697(91)90120-i

[B55] Cho YG Blair MW Panaud O McCouch SR Cloning and mapping of variety-specific rice genomic DNA sequences: amplified fragment length polymorphisms (AFLP) from silver-stained polyacrylamide gels Genome 1996 39 2 373 378 8984005 10.1139/g96-048

[B56] Lander ES Green P Abrahamson J Barlow A Daly MJ Lincoln SE Newburg L MAPMAKER: an interactive computer package for constructing primary genetic linkage maps of experimental and natural populations Genomics 1987 1 174 181 3692487 10.1016/0888-7543(87)90010-3

[B57] Peterson DG Tomkins JP Frisch DA Wing RA Paterson AH Construction of plant bacterial artificial chromosome (BAC) libraries: An illustrated guide J Agri Genomics 2000 5 1 100

